# Heterogeneity of symptoms and functions among women receiving chemotherapy for breast cancer in China: A multicentre, cross-sectional study

**DOI:** 10.3389/fpubh.2022.952710

**Published:** 2022-08-03

**Authors:** Tingting Cai, Tingting Zhou, Changrong Yuan, Chunfang Yu, Feixia Ni, Zhiren Sheng

**Affiliations:** ^1^School of Nursing, Fudan University, Shanghai, China; ^2^Department of Hematology, The Second Affiliated Hospital of Guilin Medical University, Guangxi, China; ^3^Nursing Department, The Affiliated Hospital of Medical School of Ningbo University, Zhejiang, China

**Keywords:** breast cancer, chemotherapy, heterogeneity, latent class analysis, patient-reported outcomes

## Abstract

**Background:**

Currently, few studies have explored the heterogeneity of symptoms and functions in patients with breast cancer. This study aimed to identify the subgroups of symptoms and functions in women receiving chemotherapy for breast cancer and determine whether the subgroups differed in demographic and clinical characteristics.

**Methods:**

A cross-sectional multicenter survey involving five hospitals in Zhejiang, Shanghai, Shandong, and Guangxi provinces of Mainland China was implemented between August 2020 to December 2021. Participants completed questionnaires that included the PROMIS-57, PROMIS cognitive function short form, and demographic and clinical characteristics. Latent class analysis was performed, followed by chi-square test and analysis of variance. Subsequently, significant variables were included in multinomial logistic regression.

**Results:**

A total of 1,180 patients were investigated, with an average age of 48.9 years. Three classes were identified: low symptom burdens and functions group (26.2%, Class 1), moderate symptom burdens and functions group (16.9%, Class 2), and low symptom burdens and high functions group (56.9%, Class 3). Compared with patients in Class 1 and 3, those in Class 2 consistently showed a higher tendency of having urban employee health insurance (odds ratio = 2.506, *P* < 0.05) and rural health insurance (odds ratio = 2.207, *P* < 0.05). Additionally, patients in Class 2 tended to be in their fourth cycle of chemotherapy. However, receiving chemotherapy and surgery increased the likelihood of belonging to Class 1.

**Conclusions:**

A high proportion of patients experienced varying degrees of symptom and function issues, suggesting that attention is warranted for women with breast cancer undergoing chemotherapy. Patients with the urban employee basic medical system, the new rural cooperative medical system and in the early stage of chemotherapy cycles were more likely to have symptom burdens. Middle-aged postmenopausal women reported varying degrees of cognitive issues. Additionally, surgery increased the presence of potential long-term effects in functional levels.

## Introduction

Breast cancer may present with persistent and distressing symptoms caused by disease and treatment (1, 2). For example, anticipating chemotherapy can be a stressful experience accompanied with multiple symptoms and dysfunction (3–5). Considerable evidence has shown that fatigue, pain, sleep disturbance, anxiety, and depression often manifest as a symptom cluster in women with breast cancer (6–8). Symptom clusters are sets of interrelated symptoms that share a common etiology or variance, and might cause more impact on health outcomes when compared with isolated symptoms (9). In contrast to symptoms of patients without cancer diagnosis, symptom clusters are relatively more severe and are closely related to chronic syndrome (10). Additionally, physical, social, and cognitive impairment are commonly reported in the course of chemotherapy, and can be the consequence of symptom clusters (11, 12). As the crux of patient-reported outcomes, perception of symptoms and dysfunctions prompt individuals to obtain medical treatment and care. Given that many patients experience symptoms and function issues during chemotherapy, a comprehensive approach to identify high-risk subgroups and address these care needs should be implemented.

Assessment of symptoms and functions can be obtained directly from the patients' perspective. The Patient-Reported Outcomes Measurement Information System (PROMIS) is an initiative to promote standardization and validation of multiple self-reported outcome measures across different health conditions (12). PROMIS measures are calibrated based on the item response theory model, which makes it possible to quantify a health domain based on its severity. Therefore, PROMIS measures are ideal instruments for reporting symptoms and functions.

Some efforts have been made to explore the symptom experiences of patients with breast cancer. However, variable-centered methods might dilute the heterogeneity by using total scores of the symptoms for evaluation purposes. Latent class analysis (LCA) is a commonly used person-centered approach to distinguish heterogeneous entities compared with a variable-centered method (13). It allows inter-individual variability, identifies the class-specific probability of response profiles and characteristics, and assigns similar individuals into corresponding subgroups (13). Therefore, LCA is useful for clinical and research purposes, as it elaborates the inter-individual relationship and enables health professionals to perform targeted guidance and interventions for specific groups. The mean age of diagnosis for breast cancer is 45–55 years in China, which is almost 10 years younger than the average for western patients (14). Additionally, middle-aged women accounted for a large proportion of patients with breast cancer in China (14). Given no prior attention has been given to heterogeneity for symptoms and functions in women receiving chemotherapy for breast cancer in China, it is important to understand distinct clusters and predict high-risk individuals with symptoms and function issues. Additionally, few study have adopted PROMIS measures in patients with breast cancer to identify their symptoms and functions experiences, especially in those receiving chemotherapy. Therefore, the purpose of this study was to examine the heterogeneity of symptoms and functions using the LCA approach and delineate the class characteristics using PROMIS measures in women receiving chemotherapy for breast cancer in China.

## Methods

### Study design and samples

A cross-sectional research design, which adhered to the STROBE guidelines, was adopted. Using convenience sampling method, patients with breast cancer who were hospitalized in five tertiary hospitals in Zhejiang, Shanghai, Shandong, and Guangxi provinces were recruited from August 2020 to December 2021. The inclusion criteria for participants were as follows: women aged 18 years old or above, diagnosed with breast cancer, receiving chemotherapy, and were able to write or speak Chinese. Patients who were in critical or terminal condition, had other cancers or serious diseases, refused to participate, and had psychiatric illnesses were excluded. More than 50 samples are recommended for LCA for each subgroup (13). Therefore, we included more than 1,000 patients to ensure the accuracy of the classification.

### Measures

#### Demographic and clinical characteristics

Demographic data included age, marital status, children, educational level, employment status, annual household income, and medical insurance. The health condition characteristics were cancer stage, therapeutic regimen, and chemotherapy cycle.

#### PROMIS-57

PROMIS-57 is a commonly used instrument to assess multidimensional symptoms and functions (15). The instrument consists of seven domains, including physical function (eight items), anxiety (eight items), depression (eight items), fatigue (eight items), sleep disturbance (eight items), pain interference and intensity (eight items for pain interference and a single item for pain intensity), and ability to participate in social roles and activities (eight items) (16). The total scores range from 8 to 40 and all items are rated on a five-point Likert scale, except for pain intensity, which is assessed using a numeric rating scale ranging from 0 to 10. Raw scores of all domains are transformed into T-scores, with a mean of 50 and a standard deviation of 10, indicating the reference level of the general US population (http://www.healthmeasures.net). A higher score indicates a higher level of the trait being measured, i.e., either higher degrees of function or worse symptom burden. Therefore, higher scores for anxiety, depression, fatigue, and sleep disturbance domains reflect more symptom severity. By contrast, higher scores for physical function and ability to participate in social roles and activities domains reflect better functioning (15). The psychometric properties of the PROMIS-57 had been validated in multiple studies on patients with chronic diseases including breast cancer (15, 16). The Cronbach's α of the PROMIS-57 ranged from 0.85 to 0.95 in this study.

#### PROMIS cognitive function short form

PROMIS cognitive function short form is a self-reported instrument of perceived cognitive difficulties (17). The instrument consists of four items, which are rated on a five-point Likert scale during the previous 7 days (1 = very often to 5 = never). Raw score of the four items ranges from 4 to 20 and is transformed to *T*-scores (*M* = 50, SD = 10), and lower scores indicate greater subjective cognitive difficulty (17). The Cronbach's α of the scale was 0.93 in this study.

### Procedures

Ethical approval of this study was obtained from the Institutional Review Boards of Fudan University Cancer Hospital (no 1810192-22) and Fudan University Zhongshan Hospital (no 2020-076R). The multicenter survey was supported by a mobile application named “intelligent intervention system for chronic disease follow-up,” which aimed to combine intelligent follow-up and intervention for medical staff and was available for symptom management, follow-up, health education, and visit notice. The application had been utilized to maintain long-term contact between patients and medical staff and improve patients' compliance and treatment experience in clinical settings in Affiliated Hospital of Medical School of Ningbo University for years. In this study, demographic and clinical characteristics, PROMIS-57, and PROMIS cognitive function short form were incorporated into the application.

To ensure standardization of data collection, all research assistants were previously trained to use the application. All patients completed written informed consent forms that assured the voluntariness and confidentiality of participation and their right to withdraw from the study without any penalty. Patients were also required to click on the “agree” button in the application before completing the survey if they agreed to participate. The research assistants verified the clinical characteristics of the patients to ensure consistency of the results with their medical records, and clarified any questions raised by the participants.

### Statistical analysis

Data were analyzed using SPSS version 25.0 and Mplus version 8.0. Frequencies and percentages were adopted to describe demographic and clinical characteristics. Means and standard deviations were used to delineate subscale scores.

*T*-scores of the PROMIS-57 and PROMIS cognitive function short form were dichotomized as 0 or 1 according the cutoff score for clinical difference according to PROMIS guidelines (https://www.healthmeasures.net/). Subsequently, LCA from one to six classes was performed to identify the subtypes in terms of symptoms and functions. For model selection, low Akaike information criterion (AIC), Bayesian information criterion (BIC), and sample size-adjusted Bayesian information criterion (aBIC) were used to assess information criterion. Entropy was utilized to evaluate the accuracy of classifications. Additionally, Lo-Mendell-Rubin likelihood-ratio test (LMR-LRT) and bootstrap likelihood-ratio test (BLRT) were also performed (18). Lower AIC, BIC, aBIC, larger entropy, and statistically significant LMR-LRT and BLRT were indicators of good model fit (13). Subsequently, the difference in demographic and clinical variables among the subgroups was analyzed using a chi-square test and analysis of variance.

After best-fitting models were determined, multinomial logistic regression analysis was conducted to examine whether the above-mentioned significant variables had mutually adjusted associations with latent class membership. Odds ratios (ORs) and 95% confidence intervals were calculated. Statistical significance was set at *P* < 0.05.

## Results

### Sample characteristics

Of the 1,350 participants, 170 were excluded due to missing items. A total of 1,180 patients were finally included in the final analysis. The mean age of the patients was 48.9 years (SD = 10.1, ranging from 24 to 85). Furthermore, 92.5% of them were married, and 54.5% had one child. Most participants had more than secondary school education: 19.5% received a high school diploma and 27.3% had completed a university degree or above. Most of the patients (60.5%) had an annual household income of ≥¥60,000 (approximately USD $9,240) and had urban employee health insurance (71.0%), and were unemployed (37.5%). With regard to clinical characteristics, most of them were in the early stage of cancer (37.2%). The majority received chemotherapy and surgery (39.8%) and had undergone more than five cycles of chemotherapy treatment (50.8%).

### Classification of symptoms and functions

The goodness-of-fit index comparison is listed in Table 1. As more classes were included, the values of AIC, BIC, and aBIC decreased. LMR-LRT and BLRT results favored the three-class model over the higher-class models. The three-class model also obtained the highest entropy value. Thus, based on model fit indices and consideration of clinical interpretability, a three-class model was determined to be the optimal solution.

**Table 1:** Latent class model fit comparison.

**Model**	**AIC**	**BIC**	**aBIC**	**Entropy**	**LMR**	**BLRT**	**Class probability**
1	59,939.77	60,381.15	60,104.80	—	—	—	1
2	46,625.86	47,513.69	46,957.82	0.98	<0.001	<0.001	0.43/0.57
3	41,964.12	43,298.39	42,463.01	0.98	0.03	<0.001	0.26/0.17/0.57
4	39,982.15	41,762.87	40,647.96	0.97	0.73	0.52	0.11/0.25/0.40/0.24
5	39,081.50	41,308.67	39,914.24	0.97	0.60	0.41	0.07/0.24/0.07/0.39/0.23
6	38,297.57	40,971.19	39,297.25	0.98	0.77	<0.001	0.31/0.07/0.07/0.10/0.18/0.27

**Figure 1:**
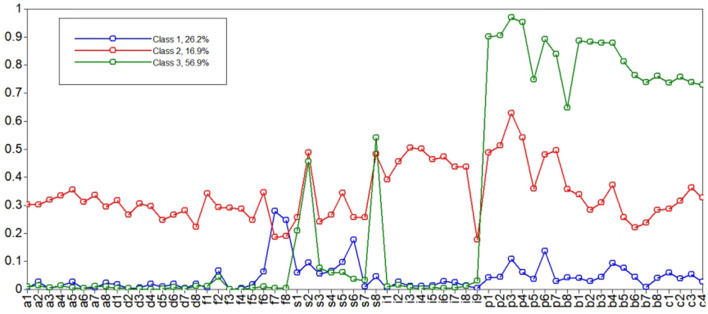
Probability of eight domains across each class.

Probability for the three-class model is shown in Figure 1. Of the 1,180 patients, 26.2, 16.9, and 56.9% were classified into one, two, and three classes, respectively. Class 1 was characterized by featuring the low probability of symptom and function domains, and was labeled “low symptom burdens and functions group.” Class 2 was labeled as “moderate symptom burdens and functions group” since patients in this group were more likely to feature moderate probability of both symptom and function domains. Class 3 was characterized by featuring the lowest probability of symptoms but the highest function domains, and was labeled “low symptom burdens and high functions group.”

Table 2 shows the scores of the eight domains in each class. Patients in Class 3 obtained the highest scores in the physical, social, and cognitive function domains. By contrast, patients with more symptom burdens were more likely to belong to Class 2.

**Table 2:** Descriptive T scores of symptoms and functions in each class.

**Domain**	**Class 1 (*n* = 309)**	**Class 2 (*n* = 200)**	**Class 3 (*n* = 671)**
Anxiety	50.96 (9.73)	61.57 (9.38)	52.97 (11.52)
Depression	51.57 (7.85)	60.12 (8.56)	49.57 (8.39)
Fatigue	50.11 (4.70)	56.03 (7.23)	46.13 (7.15)
Sleep disturbance	46.42 (7.58)	55.85 (6.91)	50.74 (6.90)
Pain interference	51.76 (7.49)	62.26 (7.53)	50.67 (7.59)
Physical function	31.04 (5.29)	40.05 (6.01)	48.71 (9.06)
Ability to participate in social roles and activities	35.61 (6.82)	44.00 (5.97)	54.43 (6.94)
Cognitive function	32.86 (7.33)	41.56 (8.42)	49.38 (8.29)

### Comparisons of demographic and clinical characteristics among the three classes

Differences in demographic characteristics among latent classes are reported in Table 3, and statistically significant differences were found in age, medical insurance, cancer stage, therapeutic regimen, and chemotherapy cycle (*P* < 0.05). These variables were included in multinomial logistic regression analysis.

**Table 3:** Differences in demographic characteristics among the latent classes.

**Items**	**Class 1 (*n* = 309)**	**Class 2 (*n* = 200)**	**Class 3 (*n* = 671)**	***P*-value**
**Age**				
20–39 years	62	28	127	0.007
40–59 years	219	154	443	
≥60 years	28	18	101	
**Marital status**				
Single	7	6	14	0.521
Married	290	181	622	
Divorced	5	6	23	
Widowed	7	7	12	
**Children**				
0	11	8	31	0.356
1	184	108	352	
2	99	67	233	
≥3	15	17	55	
**Education level**				
Primary school or below	55	42	150	0.073
Secondary school	99	66	216	
High school	50	40	140	
University or above	105	52	165	
**Annual household income**				
≤ ¥60,000 (USD $9,240)	183	135	397	0.113
¥60,001–¥120,000	89	39	194	
>¥120,001 (USD $18,516)	37	26	80	
**Medical insurance**				
Free medical insurance	0	4	3	0.003
Urban employee health insurance	137	71	241	
Urban resident basic health insurance	95	59	236	
Rural health insurance	59	50	164	
Without health insurance	18	16	27	
**Employment status**				
Employed	69	32	134	0.077
Medical leave	72	37	113	
Unemployed	98	80	264	
Retired	70	51	160	
**Cancer stage**				
I	30	12	68	0.044
II	105	63	173	
III	60	31	122	
IV	27	18	80	
Did not get precise cancer stage information yet	87	76	228	
**Therapeutic regimen**				
Chemotherapy and surgery	142	67	261	<0.001
Chemotherapy + Surgery + radiotherapy	67	55	144	
Chemotherapy + Surgery + radiotherapy + endocrine therapy	65	44	200	
Chemotherapy and other therapies	35	34	66	
**Cycles of chemotherapy**				
First	43	40	104	0.012
Second	41	26	83	
Third	41	21	72	
Fourth	44	11	54	
≥ Fifth	140	102	358	

**Figure 2:**
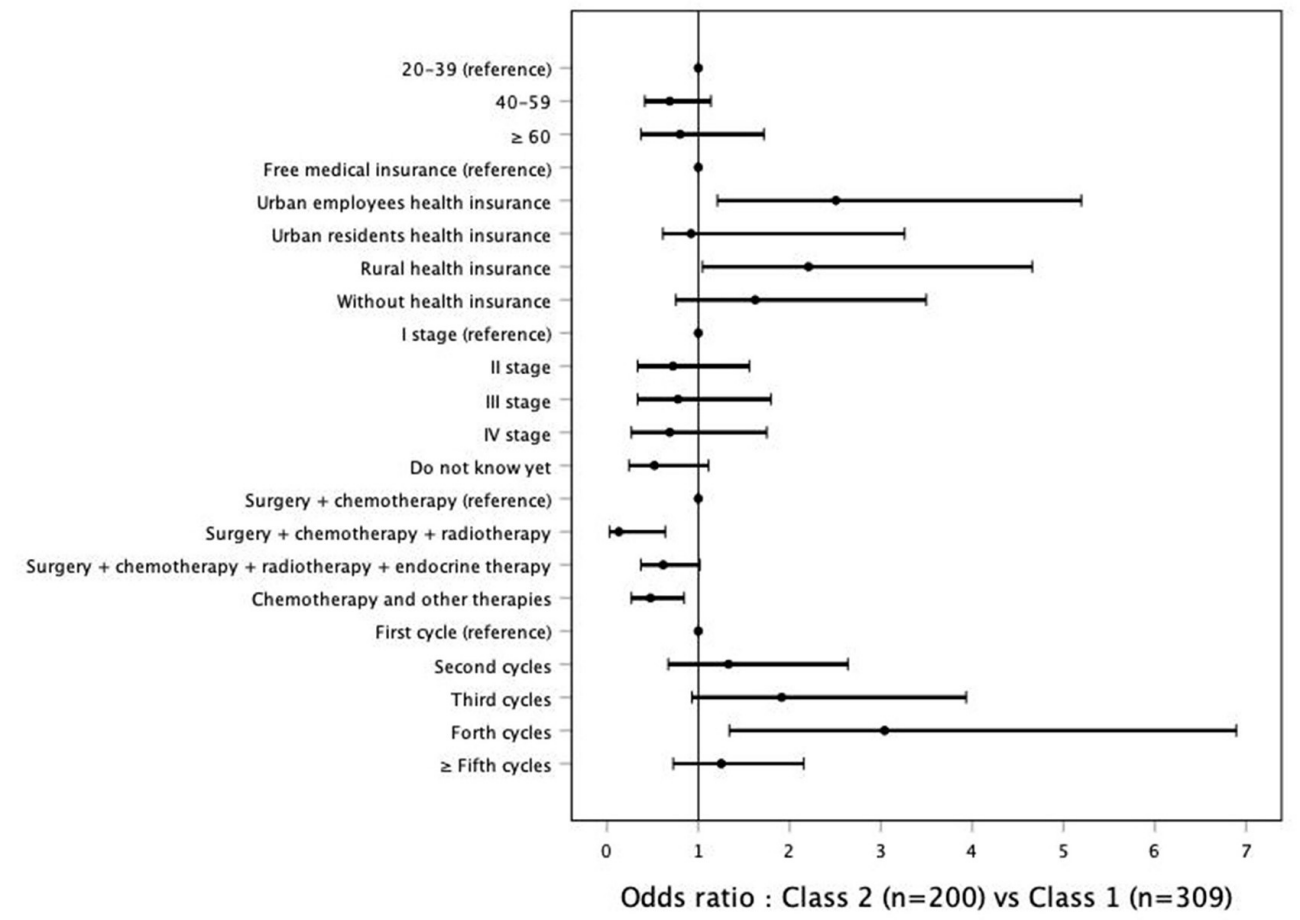
Association of demographics and clinical characteristics with Classes 2 compared with Class 1.

Multinomial regression analysis was performed with Class 1 as reference. The reference groups were 20–39 years old, free medical insurance, cancer stage I, chemotherapy and surgery, and first chemotherapy cycle. Association of demographics and clinical characteristics with Classes 2 and Class 3 compared with Class 1 were shown in Figure 2, 3. Table 4 shows that patients with urban employee health insurance and rural health insurance were at increased risk for experiencing moderate symptoms compared with those with free or no medical insurance (OR = 2.506, *P* = 0.013; OR = 2.207, *P* = 0.038). These patients were also more likely to be in their fourth cycle of chemotherapy treatment (OR = 3.040, *P* = 0.008). Patients with chemotherapy and surgery treatment were more likely to have impaired functions compared with those with other therapeutic regimens. No significant associations were found between age and cancer stage with the risk of symptoms and function issues.

**Figure 3:**
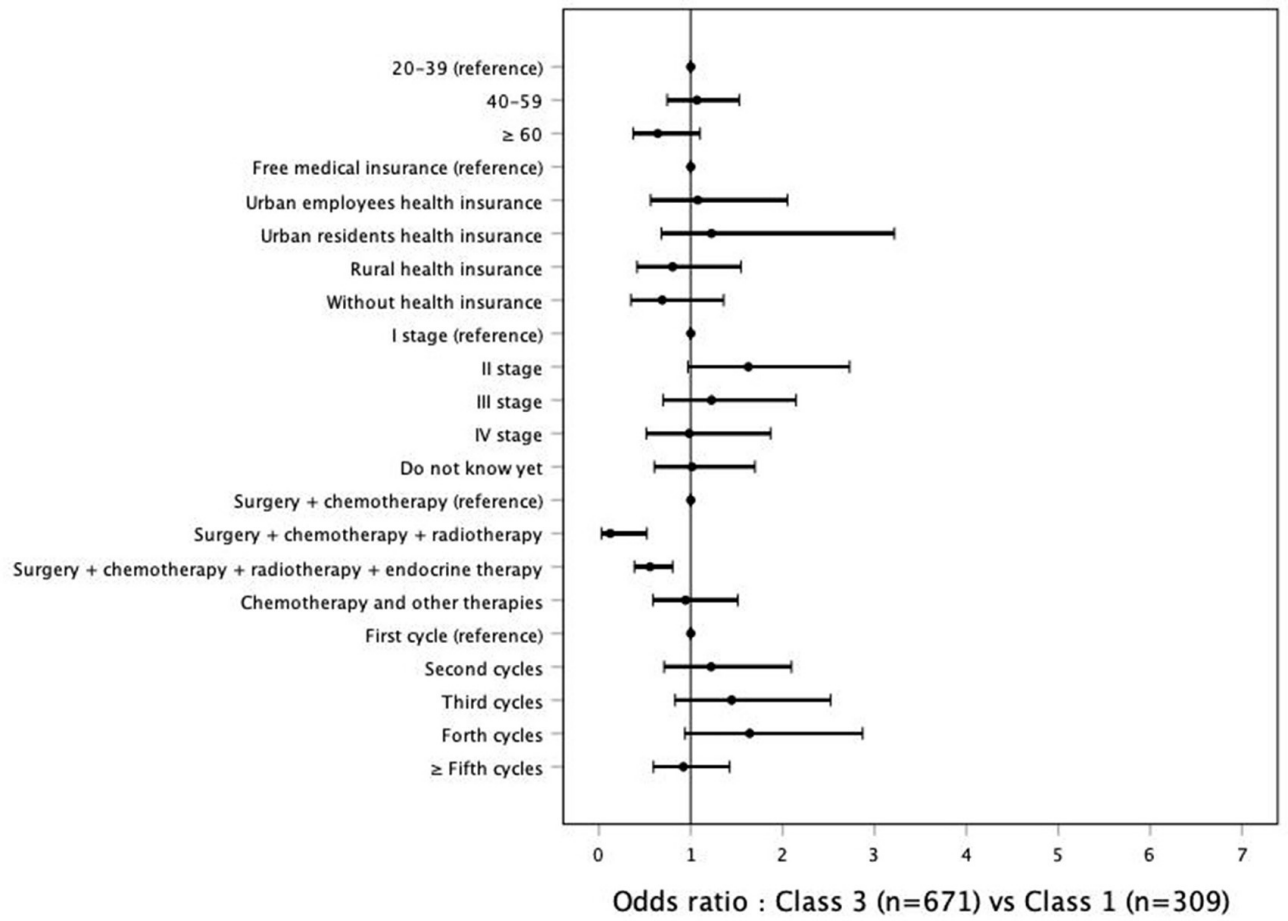
Association of demographics and clinical characteristics with Classes 3 compared with Class 1.

**Table 4:** Selected results of multinomial logistic regression: Potential prediction of latent class membership.

**Items**	**Class 2 (*****n*** = **200)**			**Class 3 (*****n*** = **671)**		
	**OR**	**95% CI**	***P*-value**	**OR**	**95% CI**	***P*-value**
**Age**						
20–39 years	Ref			Ref		
40–59 years	0.686	0.414–1.138	0.144	1.067	0.745–1.528	0.723
≥60 years	0.801	0.373–1.720	0.569	0.641	0.374–1.100	0.106
**Medical insurance**						
Free medical insurance	Ref			Ref		
Urban employee health insurance	2.506	1.209–5.195	0.013	1.076	0.564–2.051	0.824
Urban resident basic health insurance	0.921	0.613–3.260	0.166	1.225	0.680–3.217	0.369
Rural health insurance	2.207	1.046–4.657	0.038	0.802	0.416–1.546	0.510
Without health insurance	1.623	0.754–3.496	0.216	0.689	0.349–1.357	0.281
**Cancer stage**						
I	Ref			Ref		
II	0.722	0.335–1.558	0.407	1.627	0.970–2.729	0.065
III	0.777	0.336–1.797	0.555	1.226	0.700–2.147	0.475
IV	0.687	0.269–1.751	0.431	0.982	0.516–1.869	0.955
Did not get precise cancer stage information yet	0.519	0.242–1.114	0.092	1.013	0.603–1.700	0.961
**Therapeutic regimen**						
Chemotherapy + surgery	Ref			Ref		
Chemotherapy + Surgery + radiotherapy	0.130	0.026–0.637	0.012	0.123	0.029–0.522	0.005
Chemotherapy + Surgery + radiotherapy + endocrine therapy	0.615	0.372–1.017	0.058	0.556	0.386–0.802	0.002
Chemotherapy and other therapies	0.476	0.268–0.845	0.011	0.944	0.589–1.514	0.812
**Cycles of chemotherapy**						
First	Ref			Ref		
Second	1.331	0.671–2.639	0.414	1.221	0.711–2.096	0.470
Third	1.913	0.931–3.934	0.078	1.445	0.829–2.521	0.195
Fourth	3.040	1.341–6.891	0.008	1.642	0.939–2.872	0.082
≥Fifth	1.250	0.726–2.153	0.420	0.920	0.594–1.424	0.708

## Discussion

This study identified the heterogeneity for symptoms and functions in women with breast cancer undergoing chemotherapy by performing a person-centered cluster statistical analysis. Three latent classes were identified: low symptom burdens and functions group, moderate symptom burdens and functions group, and low symptom burdens and high functions group. Notably, 43.14% of the participants belonged to classes with symptom or function issues, suggesting that early detection should be considered to identify patients at risk and provide patient group-specific interventions.

Consistent with the findings of Kyranou and Li et al. (19, 20), the present study found that the symptom cluster showed similar trends of severity, especially in anxiety and depression, fatigue, and sleep disturbance domains. Although not always consistent with the tendency of symptoms, a consistent pattern was found for the function domains (21, 22). Patients in Class 3 were more likely to report low levels of symptoms but high function and were a relatively small burden group, accounting for the largest proportion. Although the patients in Class 1 had low symptoms, they had impaired functions physically and socially. In contrast, the patients in Class 2 were more concerned with their symptoms. Therefore, nurses should prioritize assessment and interventions to patients in Class 1 and 2.

The type of health insurance was found to be correlated with symptom and function levels. Patients in Class 2 were more likely to have urban employee health insurance and rural health insurance. Currently, China has three public health insurance systems, namely the urban resident basic medical system, the urban employee basic medical system, and the new rural cooperative medical system (23). However, the current tiered basic medical insurance scheme cannot cover all the health services. Patients with urban employee health insurance suffer from long-term follow-up costs and the loss of some employment-based insurance benefits due to the inability to work (24, 25). For patients with new rural cooperative medical insurance, their economic burden is higher than those with urban residents and urban employee medical insurance, and they have to pay for relatively high costs out-of-pocket after a cancer diagnosis. For example, the proportion of medical expenses coverage for the urban residents is 70–90% while there is only 50–60% for the rural residents (26). Additionally, patients who live in rural areas may face disproportionate financial problems due to higher transportation costs, higher rates of uninsured and other factors (26). Therefore, they have less access to symptom management and suffer from more perceived financial hardship due to the disease and the treatment, which might be the reason for more symptoms and dysfunction (26).

Consistent with the literature (26), patients in the early stage of chemotherapy cycles were more likely to be in the group characterized by higher symptom severity, and patients in Class 2 tended to be in their fourth cycle of chemotherapy treatment. Browall et al. (27) reported that the fatigue and pain symptoms significant affected functional status of patients with stage I–IIIa breast cancer at three to five cycles of their chemotherapy, and these symptoms were not major concerns after the 6 cycles. Therefore, our results were not against the broader literature.

Previous studies reported that age is an important and unmodifiable risk factor for functional status such as cognitive and physical function, and the prevalence of cancer-related cognitive impairment is high among females aged 60 years or above (28, 29). Mandelblatt et al. (30) explored trajectories of self-reported cognitive function over time in older women with breast cancer who received chemotherapy for 6 years, and found that less than one in 10 patients had a steep decline with accelerated aging for cognitive function. However, only 12.4% of the patients in this study were aged 60 years or older, and critically ill patients or patients who were unwilling to use the mobile application were excluded, which may have influenced the results and made the proportion of patients with cognitive decline or physical function smaller overall. Interestingly, middle-aged postmenopausal women in this study reported varying degrees of cognitive issues. A recent study in China suggested that electrophysiological cognitive impairments mainly occur in younger patients receiving chemotherapy for breast cancer (31). This status would last for years after the treatment, highlighting the importance of early detection and intervention for relatively young patients. Additionally, the largest proportion of middle-aged patients was found in groups with more symptom burdens. Middle-aged women take on important roles at work and in their families and may be an essential provider of family income (32). The impact of cancer diagnosis and the long-term effects of treatment such as chemotherapy can lead to functional limitations, physical and psychological symptoms in these patients. It may cause dilemma in balancing work, social activities, and family responsibilities (33). Therefore, a relatively high occurrence of symptoms in middle-aged women could be understandable.

The findings also provided valuable information in terms of cancer-treatment details. Receiving chemotherapy and surgery treatment was found to be associated with Class 1, which was a group with more dysfunction. In our study, most patients had undergone more than five cycles of chemotherapy treatment, and were not shortly after surgery, and they did not report significant symptoms in this stage. Therefore, our results suggested that surgery increased the presence of more potential long-term late effects in functional levels, and the results were not against previous studies (30, 34). Our study did not particularly investigate the effect of different surgery types in the functional status. Fontes et al. (35) investigated women with breast cancer surgery or without breast cancer, and assessed the impact of surgical treatment on physical activity, functional capacity, and quality of life in patients with breast cancer. The results indicated that patients who underwent breast reconstruction reported better physical activity and quality of life than patients who received breast-conserving surgery or mastectomy alone. Therefore, future research would benefit from considering these variables and increase the external validity of the results in this study.

Our study has several limitations. First, the cross-sectional design failed to explore the trajectory of class memberships. The results required further testing by using a longitudinal study design. Second, the patients were recruited in tertiary hospitals, which might limit the generalization of the findings in other clinical settings. More predictors that differentiate at-risk subgroups need to be investigated further.

## Conclusions

The person-centered approach used in this study identified the discrete heterogeneity of symptoms and functions, which leaned close to the natural convergence of the interaction patterns from a patient perspective and shed light on inter-group differences. Understanding how these groups vary during the chemotherapy treatment helped to identify subgroups of patients at risk for elevated symptoms and dysfunction. A high proportion of patients experienced varying degrees of these issues, suggesting that more attention is warranted for women with breast cancer undergoing chemotherapy. Patients with the urban employee basic medical system, the new rural cooperative medical system and in the early stage of chemotherapy cycles were more likely to have symptom burdens. Middle-aged postmenopausal women in China reported varying degrees of cognitive issues. Additionally, surgery increases the presence of potential long-term effects in functional levels. These patients may benefit from additional assessment and early intervention.

## Data availability statement

The raw data supporting the conclusions of this article will be made available by the authors, without undue reservation.

## Ethics statement

Ethical approval of this study was obtained from the Institutional Review Boards of Fudan University Cancer Hospital (no 1810192-22) and Fudan University Zhongshan Hospital (no 2020-076R). The patients/participants provided their written informed consent to participate in this study.

## Author contributions

TC and TZ: drafted the manuscript, contributed to the design of the study, completed the acquisition and analysis of data for the work. ChaY: contributed to the interpretation and critical revision of the manuscript. ZS, TC, and ChuY: completed the acquisition of data for the work, made the interpretation of data for the work. FN: involved in the analysis of data for the work, revised the manuscript critically for important intellectual content. ZS: contributed to the conception, design, interpretation and critical revision of the manuscript. All authors have read and approved the final manuscript.

## Funding

This study was supported by the Major Project for Science & Technology Innovation 2025 in Ningbo, China (2019B10035). The funding aided in the questionnaire collection process and open access publication fees of the manuscript.

## Conflict of interest

The authors declare that the research was conducted in the absence of any commercial or financial relationships that could be construed as a potential conflict of interest.

## Publisher's note

All claims expressed in this article are solely those of the authors and do not necessarily represent those of their affiliated organizations, or those of the publisher, the editors and the reviewers. Any product that may be evaluated in this article, or claim that may be made by its manufacturer, is not guaranteed or endorsed by the publisher.
